# Supplementary figures and images for: Echocardiographic and Electrocardiographic Determinants of Atrial Cardiomyopathy Identify Patients with Atrial Fibrillation at Risk for Left Atrial Thrombogenesis
Source: J Clin Med. 2022 Feb 28;11(5):1332. doi: 10.3390/jcm11051332 (PMC8911088; doi:10.3390/jcm11051332)

## Supplement Figure S1

**A: LA-GLS in SR**

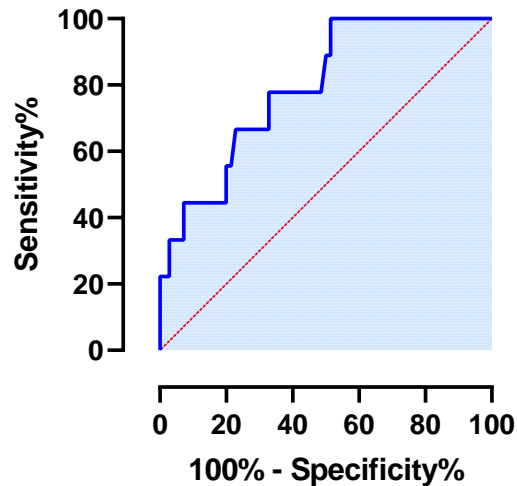

— AUC 0.79 (95%CI:0.66-0.93)

**B: LA-GLS in AF**

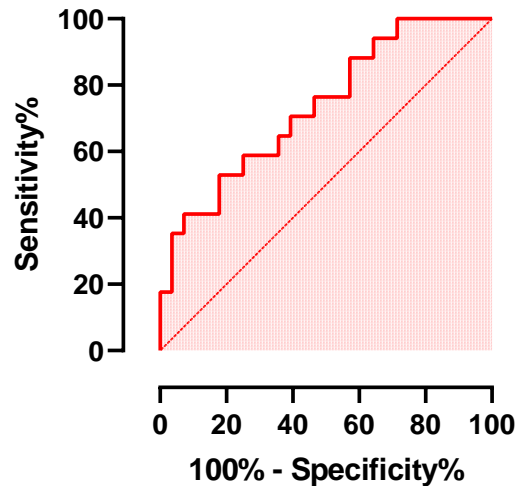

— AUC 0.74 (95%CI:0.59-0.88)

Supplement: Supplementary file 1 [file jcm-11-01332-s001.zip › jcm-1547138-supplementary.pdf]
